# High Pressure Metamorphism Caused by Fluid Induced Weakening of Deep Continental Crust

**DOI:** 10.1038/s41598-018-35200-1

**Published:** 2018-11-19

**Authors:** Bjørn Jamtveit, Evangelos Moulas, Torgeir B. Andersen, Håkon Austrheim, Fernando Corfu, Arianne Petley-Ragan, Stefan M. Schmalholz

**Affiliations:** 1 Physics of Geological Processes (PGP), The Njord Centre, Department of Geosciences, University of Oslo, P.O. Box 1048 Blindern, N-0316 Oslo, Norway; 2 Institute of Earth Sciences, University of Lausanne, Geopolis, CH-1015 Lausanne, Switzerland; 3 Centre for Earth Evolution and Dynamics (CEED), Department of Geosciences, University of Oslo, P.O. Box 1028 Blindern, N-0315 Oslo, Norway; 4 Department of Geosciences, University of Oslo, P.O. Box 1047 Blindern, N-0316 Oslo, Norway

## Abstract

Studies of mineral equilibria in metamorphic rocks have given valuable insights into the tectonic processes operating at convergent plate margins during an orogeny. Geodynamic models simulating orogenesis and crustal thickening have been constrained by temperature and pressure estimates inferred from the mineral assemblages of the various lithologies involved along with age constrains from increasingly precise geochronological techniques. During such studies it is assumed that the pressure experienced by a given rock is uniquely related to its depth of burial. This assumption has been challenged by recent studies of high pressure (HP) and ultrahigh pressure (UHP) rocks. Here, we describe an example of Caledonian HP metamorphism from the Bergen Arcs in western Norway, and show that the associated formation of Caledonian eclogites at the expense of Proterozoic granulites was related to local pressure perturbations rather than burial, and that the HP metamorphism resulted from fluid-induced weakening of an initially dry and highly stressed lower crust when thrust upon the hyperextended margin of the Baltic shield.

## Introduction

The last two decades of research have shown that prior to orogeny the lower continental crust is dry and mechanically strong^1–3^. When subject to an orogenic event, lower crustal lithologies commonly experience retrogressive metamorphism along faults and shear zones. The mineralogy of the associated product rocks reflects a large variation in temperature and pressure conditions ranging from eclogite facies to amphibolite facies assemblages^4–6^. Retrogressive metamorphism is invariably associated with the formation of hydrous minerals and often also carbonates, and both the mineralogical change and the frequently associated grain size reduction lead to a substantial reduction in rock strength. A pronounced mechanical weakening is reflected by the ductile behaviour of the shear zones in which these rocks are found. Such observations inspired early models of the lithosphere such as the ‘jelly-sandwich’ model^7^ where the lower crust is assumed to be wet and mechanically weak, and hence plate tectonic stress would be transmitted through the brittle upper crust and a strong upper mantle lithosphere.

Today, an increasing number of studies and observations indicates that structural and metamorphic transformation of initially dry lower crust involves an early stage of seismic failure^6,8–10^. Metamorphism and shear zone development then follow in the wake of lower crustal earthquakes. Hence the lower crust is subject to high differential stress prior to the production of weak rocks in shear zones^11^.

In the following, we present observations from the Bergen Arcs in western Norway where fluid induced retrogression of lower crustal granulites followed initial seismic faulting. Retrogression produced both eclogite facies and amphibolite facies lithologies, which then became loci of shear zone development. Through a simple force balance model, which is supported by 2D analytical stress-field calculations, we demonstrate that the formation of weak domains in a highly stressed lower crust will lead to significant pressure perturbations. We show that rheology-induced pressure increases to eclogite facies conditions above 2 GPa are consistent with the pressure-temperature-time history experienced by this crustal volume without invoking tectonic burial beyond ca 55 km depth. Hydration and seismic deformation continued during uplift to depths of ca 35 km, where amphibolite facies metamorphism associated with late pegmatite intrusions occurred near 600 °C at 423.6 ± 1 Ma, some 5 million years after eclogite formation.

## Geological Setting of the Bergen Arcs

**Figure 1:**
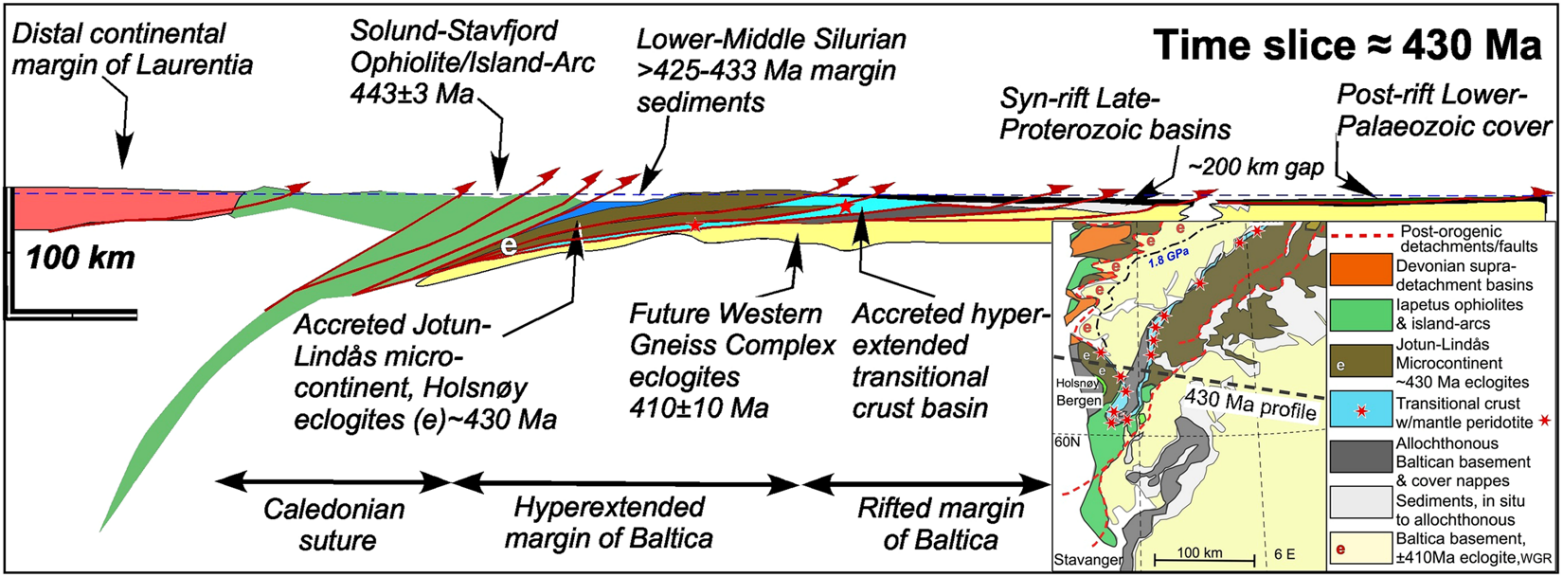
Reconstruction of the South Scandinavian Caledonides. E-W profile across the Bergen Arcs (1:1, horizontal-vertical scale) at 430 Ma, prior to the final continental Scandian collision. The leading edge of the Jotun-Lindås microcontinent is buried beneath the accreted outboard ophiolite/island arc terrains in the west, while it is thrust upon the hyperextended margin of Baltica in the east. The eclogitized and amphibolitised shear zones in the Lindås nappe formed at this stage, before the main continent-continent collision between Baltica and Laurentia. The onset of the Himalayan-type continental convergence occurred in the Lower Devonian and eventually produced regional high-to ultra-high-pressure metamorphism in the Baltic basement at 410 ± 10 Ma. A 1.8 GPa isobar is shown on the inset map.

The Lindås Nappe in the Bergen Arcs is composed of granulite facies remnants of Proterozoic lower crust belonging to the former Jotun-Lindås microcontinent (Fig. 1). Before the Caledonian plate convergence, the Jotun-Lindås microcontinent was separated from the margin of Baltica by a hyperextended domain with transitional thinned crust (<10 km), exhumed meta-peridotites, and extensional allochthons of continental crust^12^. The profile in Fig. 1 illustrates the tectonic setting in the Middle Silurian, at approximately 430 Ma. At this initial stage of the Scandian collision, ophiolites and Laurentian island-arc complexes were emplaced onto the Jotun-Lindås microcontinent and its fossiliferous Middle Silurian cover^13^. These events caused fluid-induced metamorphism transforming the 930 Ma old anhydrous granulite facies mineralogy^14^ to eclogites and amphibolites in shear zones, breccias and along fractures located near the leading edge of this microcontinent. The eclogites and amphibolites are now exposed on Holsnøy Island (Fig. S1). The hyperextended Baltic margin closed during the collision, which continued into the Lower Devonian and resulted in large-scale nappe translation (>600 km) onto Baltica and formation of a Himalayan-type mountain belt. The regional (U)HP metamorphism and metamorphic zonation in the Western Gneiss Complex at 410 ± 10 Ma (see inset map in Fig. 1) formed during the terminal stages of the collision^15,16^.

On the island of Holsnøy (Fig. S1b), pseudotachylytes, fine-grained or glassy fault rocks believed to reflect earthquake related frictional melting, are abundant on faults where the granulite facies rocks experienced Caledonian metamorphism. Such faults show single rupture displacements of up to 1.7 m^10^. The fault wall rocks display intense fragmentation often without significant shear strain, followed by healing processes through grain growth and formation of eclogite or amphibolite facies minerals, including hydrous phases such as amphibole, micas and clinozoisite^8,17^. Infiltration of hydrous fluids was thus directly associated with the seismic slip.

A significant rheological weakening associated with formation of the fine-grained and hydrous eclogites and amphibolites led to development of ductile shear zones in areas initially deformed by brittle failure (Fig. S2a,b). Relict pseudotachylytes can locally be observed ‘floating’ in the shear zones, providing unambiguous evidence for ductile deformation predated by brittle failure of granulite facies rocks^10^. All rock units are locally cut by pegmatitic veins (Fig. S2c,e). When these cut the original granulite, they are often rimmed by a cm to dm wide selvage of amphibolite facies rocks (Fig. S2d) attesting to the introduction of hydrous fluids and associated retrograde metamorphism.

## Conditions and Timing of Shear Zone Development

Early estimates of the conditions of eclogitization in shear zones were in the range 650–750 °C and 1.5–2.1 GPa^18,19^, while late amphibolite facies retrogression were reported to occur at significantly lower pressures at ca 600 °C and 0.8–1.0 GPa^19^. Recently, Bhowany *et al*.^20^, reported that seismic faulting occurred near 680 °C, 1.5–1.6 GPa, i.e. at amphibolite facies conditions where plagioclase was still stable. This is consistent with a detailed description of fault wall rock alteration reported by Petley-Ragan *et al*.^17^. Peak eclogite facies metamorphism, however, was interpreted to occur at similar temperatures, but at significantly higher pressure (2.1–2.2 GPa^20^). This was assumed to imply continued burial of the crustal volume after the stage of seismic deformation. Finally, retrogressed eclogite was reported to form at pressures near 1.6–1.7 GPa, still at approximately the same temperature.

**Figure 2:**
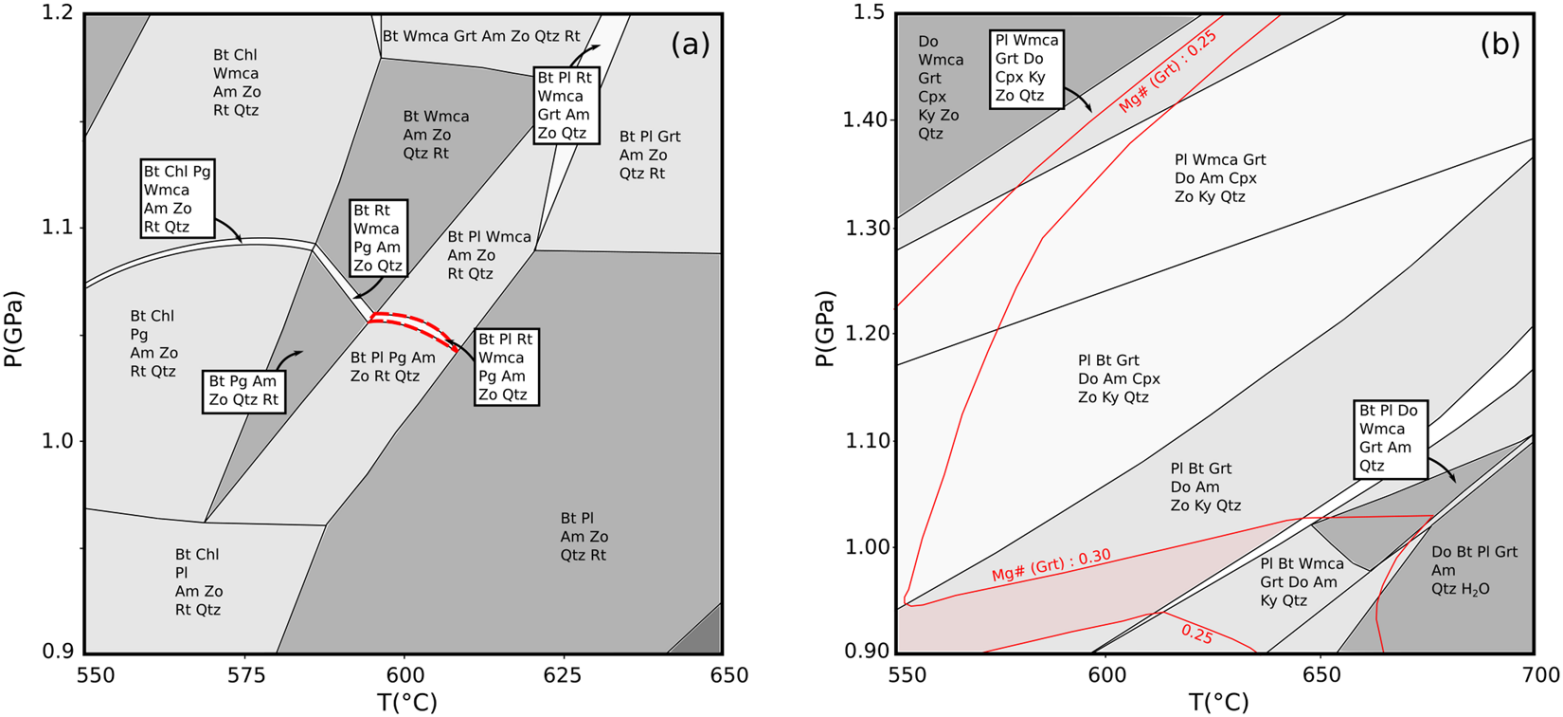
Phase diagram sections. (**a**) Pressure-Temperature section for the amphibolite sample (HA-2-17). The stability field for the observed mineral assemblage is indicated by a dashed red line. (**b**) Pressure-Temperature section for the dark homogeneous zone of pseudotachylyte shown in Fig. S4, contoured for garnet compositions (red lines). Although the latter assemblage is not very sensitive to temperature, both assemblages formed at a pressure near 1 GPa, and both could have formed at a temperature near 600 °C. See *Methods* for solid solution models and abbreviations.

To extend the results of Bhowany *et al*.^20^, we have performed phase diagram analysis (Methods) of the amphibolite facies mineral assemblage that developed around late stage feldspar rich pegmatites which cut both eclogite and amphibolite facies shear zones (Figs S2c,d and S3). In addition, we have constrained the pressure-temperature stability of mineral assemblages formed in a pseudotachylyte vein with an amphibolite facies mineral assemblage developed along a large displacement (1.7 meter) fault in an area with abundant amphibolite facies shear zones (Fig. S4). The results are shown in Fig. 2. For a pure H_2_O-fluid, the mineral assemblage in the amphibolite developed around the pegmatite is only stable in a very narrow domain of the pressure-temperature space, and puts narrow limits on the pressure and temperature conditions of formation near 600 °C and 1.05 GPa (Fig. 2a). This is in agreement with previous estimates of the conditions of amphibolitization at Holsnøy^19^. The mineral assemblage of the pseudotachylite in the large displacement fault is not very sensitive to temperature. However, both the mineral assemblage and the composition of the garnet present are consistent with formation at similar conditions near 1 GPa and 600 °C (Fig. 2b).

**Figure 3:**
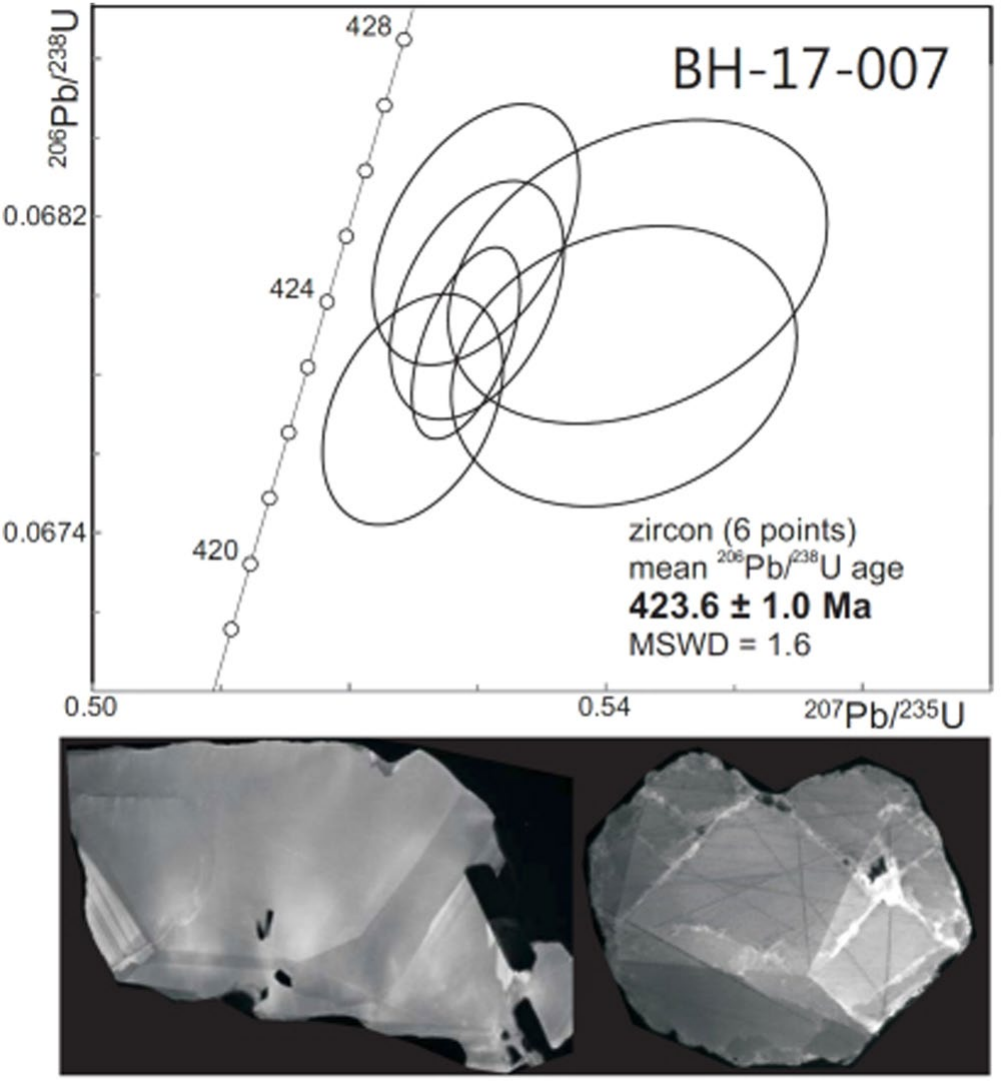
U-Pb geochronology. Top: Concordia diagram with U-Pb analyses of zircon from the pegmatite. Ellipses indicate 2σ uncertainties. Bottom: Cathodoluminescence images of typical zircons in the sample (about 100–200 μm wide). The one on the left displays an irregular outer shape, internal regular growth zoning and some feldspar inclusions (in black), the one to the right shows minor straight zoning and a network of fractures healed by new zircon.

To constrain the age of the latest stage of amphibolitisation, U-Pb geochronology (see *Methods* for analytical details) was carried out on an albite-, muscovite-, biotite-, kyanite-, amphibole-bearing pegmatite. These are common at Holsnøy and crosscut both eclogite- and amphibolite facies shear zones. Zircon is relatively abundant in the sample, along with rutile and apatite. It occurs as colourless, equant fragments, locally exhibiting crystal faces, and in minor amounts as euhedral, equant to very short prismatic grains. Inclusions of other minerals, mainly feldspar, are common. Cathodoluminescence images reveal a faint but distinct regular growth zoning and also local zones of reworking with fracturing and recrystallization or overgrowths (Fig. 3).

The zircons are extremely low in U, ranging from 1.2 to 3.1 ppm, and Th/U ~ 0 (Table S1). Consequently, only little Pb was available (<0.2 ppm) for the measurements and initial attempts using single grains were not successful. Six analyses based on fractions of 3 to 8 grains are clustered to the right of the Concordia curve. Given the low amount of U, and the fact that they all underwent chemical abrasion, it is unlikely that the discordance is due to Pb loss. It may instead be the result of small excesses of initial ^231^Pa. The analyses yield overlapping ^206^Pb/^238^U ages with an average of 423.6 ± 1.0 Ma (Fig. 3). This pegmatite age overlaps with previous age determinations on eclogites from Holsnøy by Glodny *et al*.^21^(422 ± 10 Ma by the Sm-Nd methods, and 425 ± 4 by the Rb-Sr method), and Bingen *et al*.^22^, (423 ± 4 by U-Pb), but is slightly younger than the U-Pb age obtained by Glodny *et al*.^23^ (429 ± 3 Ma). The amphibolite facies metamorphism has previous been assumed to occur at 410–415 Ma based on Rb-Sr data^23^. An older U-Pb age (427.4 ± 0.9 Ma) from an amphibolite facies assemblage^23^ was interpreted to represent inherited zircons from eclogite facies rocks. Our new data for the last phase of amphibolitisation indicate that the age of the lower-pressure assemblage was much closer to the high-pressure stage than previously assumed, possibly nearly coeval. However, our phase diagram analysis indicates that the amphibolite facies assemblages that developed around the late pegmatites formed at 50–100 °C lower temperatures than the eclogites and thus appear to have formed later, after a period of pressure reduction and cooling.

## Weakening Induced Pressure Effects

**Figure 4:**
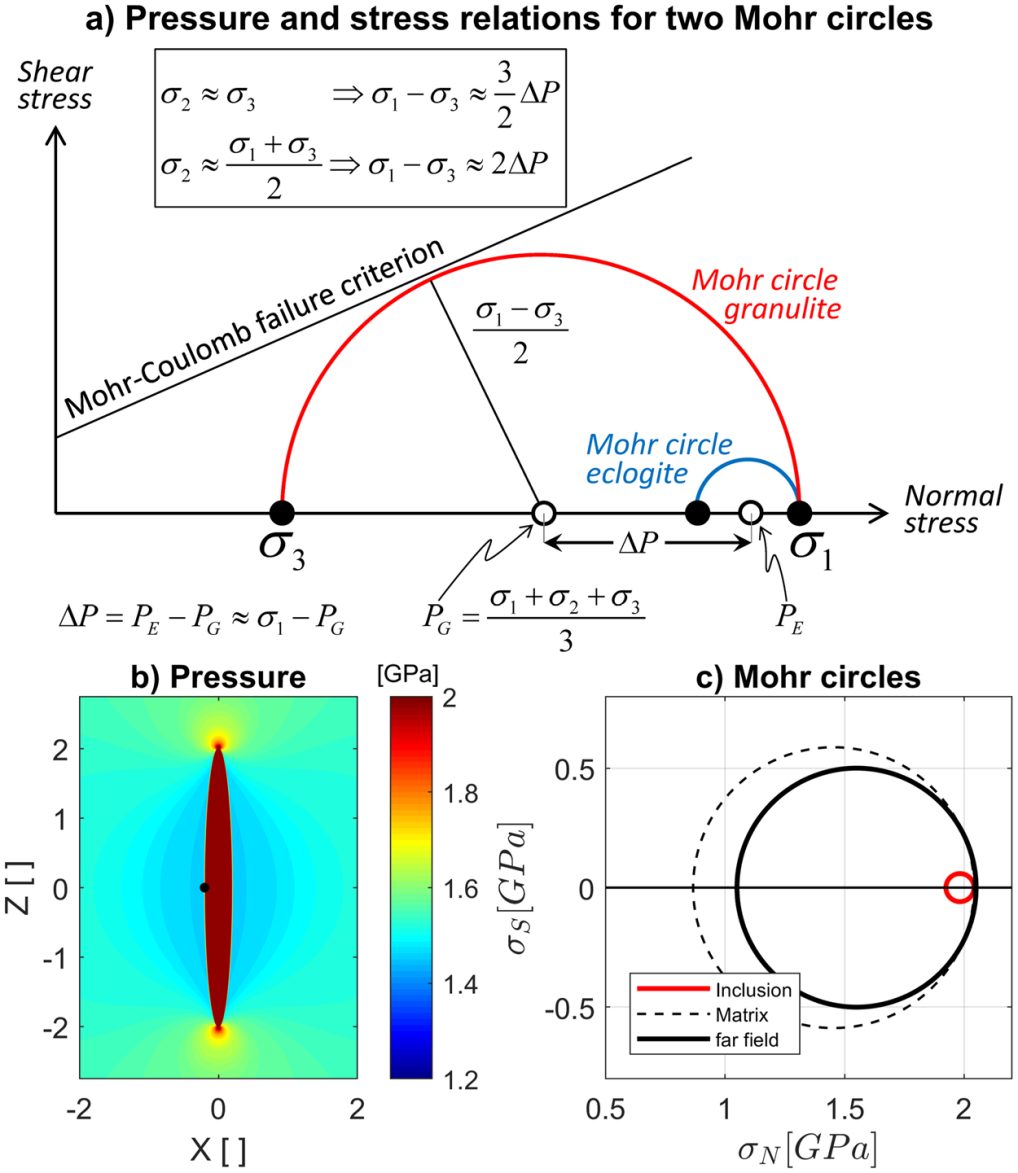
Pressure and stress relations. (**a**) Representative Mohr circles for the strong granulite and the embedded weak eclogite. For the configuration shown in **b**), σ_1_ in the granulite and eclogite are identical at their interface. Pressure in the granulite, P_G_, is different from pressure in the eclogite, P_E_. Formulas show approximate relations between differential stress in granulite, σ_1_–σ_3_, and pressure variation, ΔP, for small differential stress in the eclogite and two possible values for the out-of-plane principal stress, σ_2_. (**b**) Analytically calculated pressure field for a weak elliptical inclusion in a strong matrix (analytical solution from Moulas *et al*.^25^). Spatial coordinates are dimensionless, since result is scale independent, and pressure has been calculated for a far-field differential stress (compression parallel to X-direction) of 1 GPa in the matrix and a pressure of 1.55 GPa (far-field Mohr circle in **c**)). The strength of the inclusion is ten times less than that of the matrix. (**c**) Analytically calculated Mohr circle for the inclusion (red) and matrix (black dashed) at the contact (black circle in **b**)), and Mohr circle for the far-field compressional differential stress (black).

Thermo-mechanical numerical simulations of continental lithosphere shortening showed that the development of a weak shear zone within a stressed lower crust can generate a significant pressure increase inside the shear zone as the corresponding differential stress decreases^24^. This pressure increase results from the force balance across the shear zone and can be explained with a conceptual Mohr circle diagram that is based on a 2D analytical solution for a stressed body, or matrix, with a weak elliptical inclusion (Fig. 4; Moulas *et al*.^25^). The inclusion mimics the shear zone. The force balance between inclusion and matrix requires that the total stress normal to the inclusion-matrix interface must be identical in the inclusion and the matrix (Fig. 4a). The total stress is the sum of the pressure and the deviatoric stress. To illustrate the stress state, we consider a configuration where there is only normal stress acting on the inclusion-matrix interface and the shear stress is zero (Fig. 4a and black dot in b). For such configuration the deviatoric stress is half the differential stress. Since the deviatoric stress is smaller in the weak inclusion than in the strong matrix, the pressure in the weak inclusion must be correspondingly larger than the one in the matrix to obtain the same total stress (Fig. 4). The pressure variation depends on the aspect ratio of the inclusion and its orientation with respect to the applied differential stress^25^. Thus, some weak inclusions will develop a significant pressure increase, while others will not. The analytical results are valid for both viscous and elastic materials (Fig. 4b and c; Moulas *et al*.^25,26^).

When applied to the observed eclogite shear zones in the Bergen Arc granulites, the above mechanical models can explain the recorded pressure variations in the following steps: First, the granulite is under sufficient differential stress to cause seismic failure (≥1 GPa^10^). Second, fracturing of the granulite allows inflow of fluids. Third, the fluids cause metamorphic reactions and the reacting granulite weakens mechanically. The geometry of the weakened regions is controlled by the geometry of the fractures and can be described as elliptical regions in 2D (Fig. 4b). Depending on the orientation and aspect ratio of the weakened regions (see Moulas *et al*.^25^) the pressure can rise locally. The magnitude of the pressure rise is evaluated in the following (Fig. 4b and c).

The absolute magnitude of the pressure in the granulite is ${P}_{G}=\frac{{\sigma }_{1}+{\sigma }_{2}+{\sigma }_{3}}{3}$, where *σ*_1_, *σ*_2_ and *σ*_3_ are the principal stresses. The differential stress, *σ*_1_ − *σ*_3_, in the weak product rocks will be significantly smaller than in the strong granulite, and the pressure in the product (*P*_*E*_) is close to the maximal principal stress, *σ*_1_, in the granulite (Fig. 4a). The force balance requires that the two Mohr circles for granulite and weak product rocks must touch at one point. In the presented example both Mohr circles touch at the main principal stress (*σ*_1_) of the granulite. The value of *σ*_1_ − *σ*_3_ in the granulite required to cause a pressure perturbation, ${\rm{\Delta }}P={P}_{E}-{P}_{G}\approx {\sigma }_{1}-{P}_{G}$, between granulite and eclogite depends on the value of *σ*_2_, the principal stress in the third, out-of-plane direction. Assuming ${\sigma }_{2}\approx {\sigma }_{3}$ provides the relationship

$${\sigma }_{1}-{\sigma }_{3}\approx \frac{3}{2}{\rm{\Delta }}P.$$

Assuming $${\sigma }_{2}\approx ({\sigma }_{1}+{\sigma }_{3})/2$$ yields

$${\sigma }_{1}-{\sigma }_{3}\approx 2{\rm{\Delta }}P.$$

Hence, a pressure perturbation of 0.5 GPa requires a differential stress between 0.75 and 1.0 GPa depending on *σ*_2_ (Fig. 4b and c). These differential stresses approach the frictional strength of compressed continental rocks at depths between 20 and 30 km (e.g. Kohlstedt *et al*.^27^). Rock deformation experiments suggest that the transition from semi-brittle deformation to fully plastic (or viscous creep) deformation occurs when the differential stress is equal to the effective confining pressure of the rock (the so-called Goetze’s criterion^27^). Assuming that the effective confining pressure in the granulite is close to the metamorphic pressure estimate for the recrystallized pseudotachylites (1.5 GPa^20^), indicates that differential stresses exceeding 1.0 GPa for semi-brittle deformation are feasible in the granulite. Hence, both the rock’s frictional strength and the stress magnitudes required for the onset of plastic deformation support differential stresses near 1.0 GPa in the granulite. It is thus feasible to generate pressure perturbations exceeding 0.5 GPa in the weak rock when embedded in a strong granulite under high differential stress. The pressure difference between the mineral assemblage described as recrystallized pseudotachylite (1.5–1.6 GPa^20^) and the peak eclogite facies metamorphism (2.1–2.2 GPa^20^) may thus be due to the excess pressure generated by weakening of shear zone rocks following hydration without the need of further burial to >70 km depth.

## Discussion

In the tectonic scenario presented in Fig. 1, Caledonian eclogite-forming metamorphism of the Proterozoic granulites of the Bergen Arcs took place as the Jotun-Lindås nappe was thrust upon the hyperextended margin of Baltica and contemporaneously overthrust by nappes of oceanic affinity (Fig. 1). In this scenario, the rocks in which amphibolites and eclogites formed at 423–429 Ma, were probably never buried to Himalaya-like depth (twice the normal continental crust) and never experienced conditions corresponding to lithostatic pressures above ca. 1.5 GPa. This is furthermore broadly consistent with the density of fluid inclusions occurring in felsic extension veins interpreted to have formed during eclogitization^28^.

Mineral assemblages indicate an isothermal pressure increase of >0.5 GPa following seismic failure^20^ and a subsequent decrease by ca. 1.0 GPa towards amphibolite facies conditions (Fig. 2). Assuming an average rock density of 2900 kg/m^3^ a lithostatic pressure >0.5 GPa corresponds to a depth >18 km from an initial depth of ca 50–55 km. This is not consistent with the tectonic scenario shown in Fig. 1. Moreover, the lowest thermal gradients considered reasonable for subduction zones are ca. 5 °C/km (e.g. Syracuse *et al*.^29^; Penniston-Dorland *et al*.^30^). Hence, a >18 km depth increase by burial due to subduction should correspond to a temperature increase of >90 °C. Therefore, an isothermal pressure increase of >0.5 GPa due to burial by subduction is thermo-mechanically not feasible and calls for alternative mechanisms.

Moreover, the entire metamorphic history from the initiation by seismic failure and eclogite formation in shear zones to the intrusion of tonalitic pegmatites, with an amphibolite facies selvage formed at ca. 600 °C, 0.8–1 GPa, took place within 5 million years or less. Isothermal subduction from 50 to >70 km, followed by near isothermal exhumation to ca 55 km depth, and finally return to less than 35 km depth within this time interval does not seem feasible for the relevant tectonic scenario.

The alternative model that we propose here is that a local weakening of dry and highly stressed lower crust naturally leads to pressure perturbations of the magnitude observed here. This will apply to any convergent plate boundary where seismic activity and associated fluid introduction leads to weakening of lower crustal volumes^10^. The magnitude of the pressure perturbation will depend on the initial stress level, the local geometric constraints and the magnitude of the weakening. For a given field area, one would expect to see a variety of pressure conditions recorded by the more or less synchronously developed hydrated product rocks. Eclogite facies shear zones may thus exist essentially side-by-side with amphibolite facies shear zones developed at the same depth and temperature conditions.

In the Bergen Arcs case, many lines of evidence indicate that eclogite facies metamorphism is not necessarily the result of deep burial of continental material, but rather to local fluid induced weakening of initially highly stressed dry granulites. Since the lower continental crust is expected to be both dry and strong *prior to* an orogenic event^3^ and earthquake induced fluid introduction with associated metamorphic weakening processes seems to be common *during* orogeny, weakening induced pressure perturbations in the lower crust will also be common during orogenic events. This may explain a number of enigmatic observations of contrasting PT estimates from lower crustal lithologies undergoing retrogressive metamorphism.

Finally, the presence of pseudotachylytes recrystallized under pressures as low as 1 GPa, illustrates that seismic deformation was not confined to a fixed event prior to eclogite facies pressure conditions, but likely occurred throughout the metamorphic history of this lower crustal volume.

## Methods

### Calculation of P-T sections

For the calculation of the P-T section of the amphibolite we performed Gibbs free energy minimization^31,32^ using Perple_X (http://www.perplex.ethz.ch). We considered the following solution models *Bio(TCC)* for biotite^33^, *cAmph(DP)* for clinoamphibole^34^, *Mica(CHA1)* for white mica^35,36^, *feldspar* for potassium feldspar and plagioclase^37^, *Gt(GCT)* for garnet^38^, *Chl(HP)* for chlorite^39^, *Omph(GHP)* for clinopyroxene^34,40^ and *IlGkPy* for ilmenite (ideal). We considered the K_2_O-Na_2_O-CaO-FeO-MgO-Al_2_O_3_-SiO_2_-TiO_2_-H_2_O model system and the thermodynamic database of Holland and Powell^41,42^. The mineral abbreviations are after Siivola and Schmid^43^. The bulk composition that was used is (in wt%): K_2_O: 0.53; Na_2_O: 2.15; CaO: 12.68; FeO: 6.96; MgO: 8.47; Al_2_O_3_: 20.90; SiO_2_: 43.86; TiO_2_: 0.40; H_2_O: saturated.

The same method was employed for the calculation of various P-CO_2_ sections for the pseudotachylite. The different sections were calculated at 620, 650, 670, 700, 720, and 750 C. Dolomite was modelled using the oCcM(HP) solid solution^41^. The bulk composition that was used is (in wt%): K_2_O: 0.8; Na_2_O: 3.36; CaO: 10.38; FeO: 3.83; MgO: 3.84; Al_2_O_3_: 24.72; SiO_2_: saturated. The total H_2_O and CO_2_ was constrained from the amount of LOI.

#### U-Pb analysis

After crushing and various conventional concentration steps, the zircon grains were selected under a binocular microscope and subsequently subjected to chemical abrasion^44^. This was followed by spiking with a ^202^Pb-^205^Pb-^235^U tracer, dissolution, chemical isolation of U and Pb with ion exchange resin, and mass spectrometry, after the procedure of Krogh^45^ with modifications described in Corfu^46^. Most of the measurements were performed with an ion counting secondary electron multiplier, correcting the Pb fractionation with the ^205^Pb/^202^Pb ratio of the spike and U with 0.12%/amu. Blank corrections were 0.1 pg for U and ≤2 pg Pb. Any remaining initial Pb was corrected using compositions calculated with the model of Stacey and Kramers^47^. The tracer is calibrated against the ET100 solution (Condon, pers. comm). Plotting and regressions were done with the Isoplot software package^48^. The decay constants are those of Jaffey *et al*.^49^.

## Electronic supplementary material

Supplementary material

## Data Availability

All of the data used are contained within the paper.
