# Supplementary material for: High Pressure Metamorphism Caused by Fluid Induced Weakening of Deep Continental Crust
Source: Sci Rep. 2018 Nov 19;8:17011. doi: 10.1038/s41598-018-35200-1 (PMC6242872; doi:10.1038/s41598-018-35200-1)
Supplement: Supplementary file 1 — Supplementary material [file 41598_2018_35200_MOESM1_ESM.pdf]

Supplementary material

HIGH PRESSURE METAMORPHISM CAUSED BY FLUID INDUCED WEAKENING OF DEEP CONTINENTAL CRUST

Maps and field images

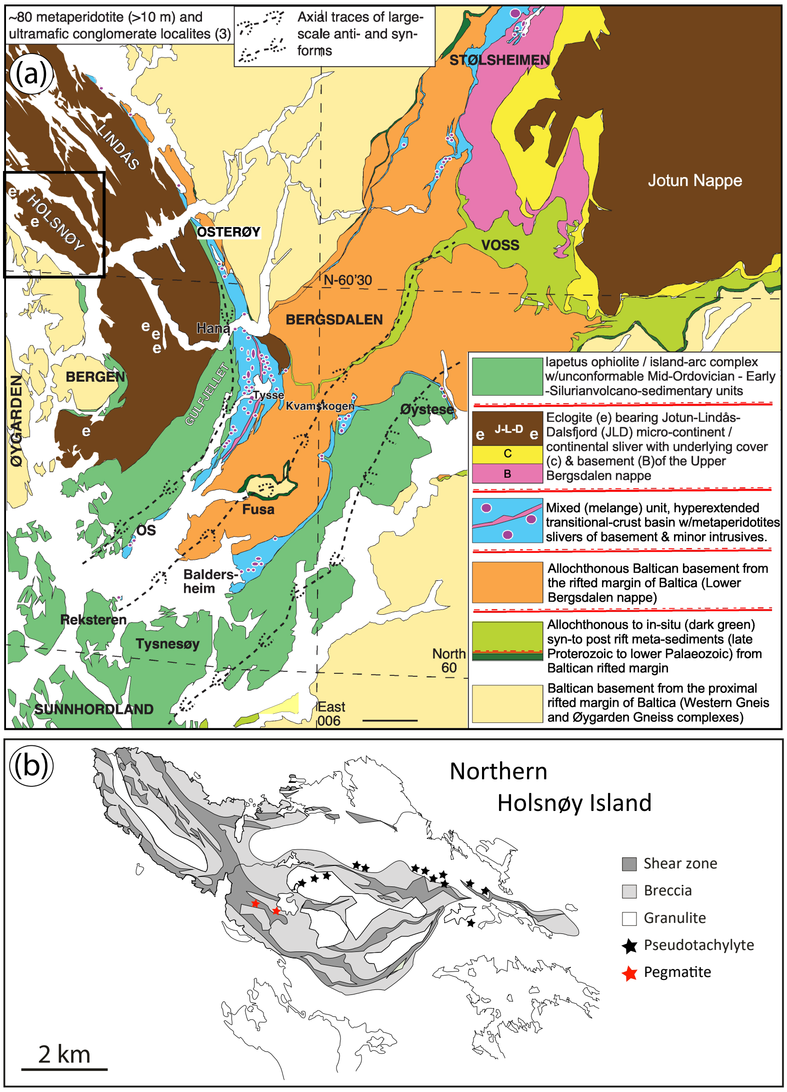

Figure S1 | (a) Simplified map of the Caledonides of the Bergen-Jotun area in western Norway. The map shows the main tectonic units and the location of the eclogite bearing rocks studied in Holsnøy and their distribution in the Lindås-Jotun Nappe Complex. (b) Map of northern Holsnøy compiled from the work of Austrheim and co-workers. Notice the distribution of eclogite- and amphibolite-facies breccias and shear zones and the common presence of pseudotachylytes. The studied pegmatites are shown by red stars.

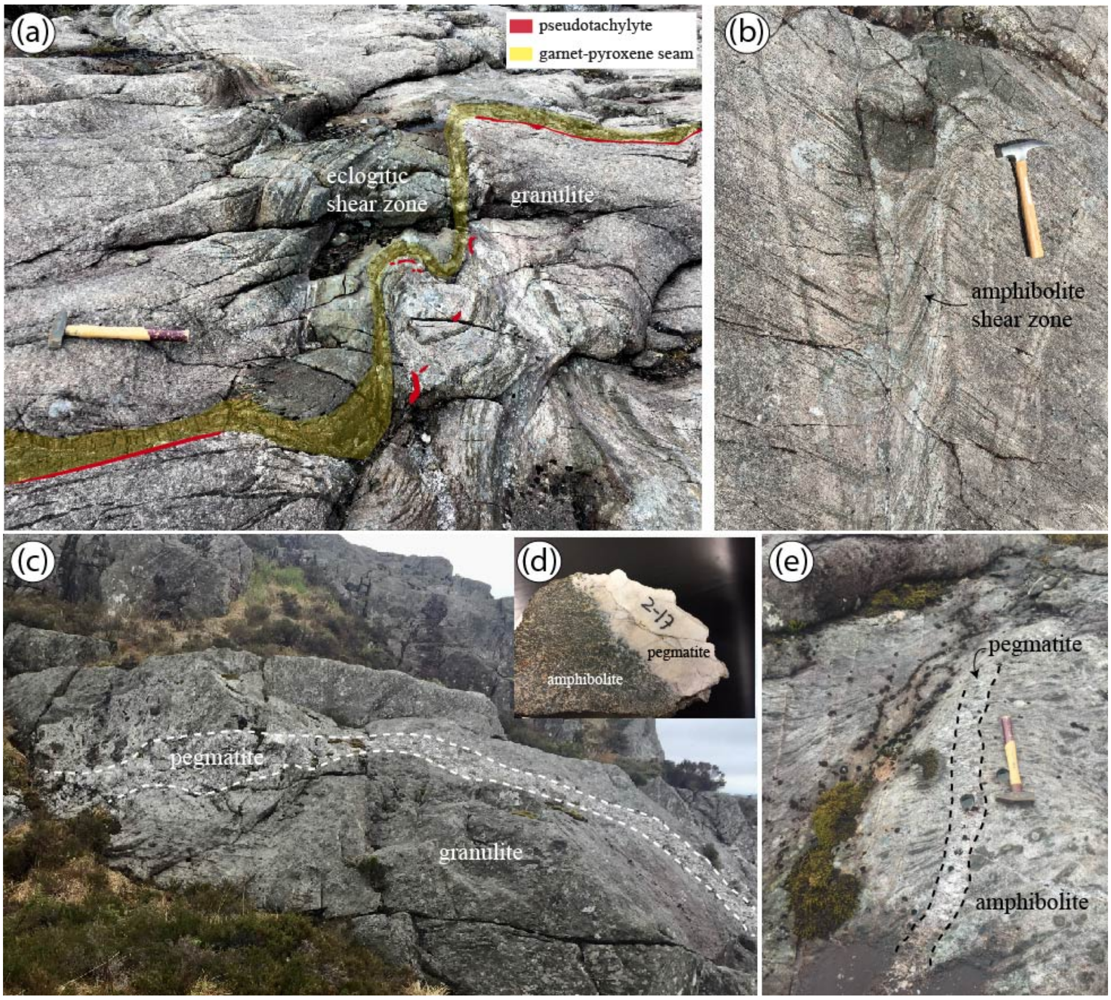

Figure S2 | Field relations illustrating initial seismic faulting, followed by development of eclogite- and amphibolite facies shear zones, and finally intrusion of albite-rich pegmatites. a) Eclogite facies shear zone with relict pseudotachylyte reflecting and early stage of brittle failure before shear zone development. b) Amphibolite facies shear zone. c) Albite-rich pegmatite crosscutting granulite. d) These pegmatites are associated with extensive amphibolitisation along their margin where they cut granulites or eclogites. e) Pegmatite crosscutting amphibolite.

Petrography and mineral compositions

The late stage amphibolite shown in Fig. S2d is composed of tschermakitic amphibole; clinozoisite (X_{Cz}=0,0.84-87); plagioclase (Ab_{24-31})(close to the pegmatite, the plagioclase is albitic); paragonite (Na/(Na+K)=0.88-0.91); phengite (6.30 Si per formula unit; K/(K+Na)≈0.80; X_{Mg}≈0.70); biotite (X_{Mg}=0.76-0.78); quartz, rutile and magnetite. Chlorite forms as a secondary phase after biotite. Representative microstructures are shown in Fig. S3.

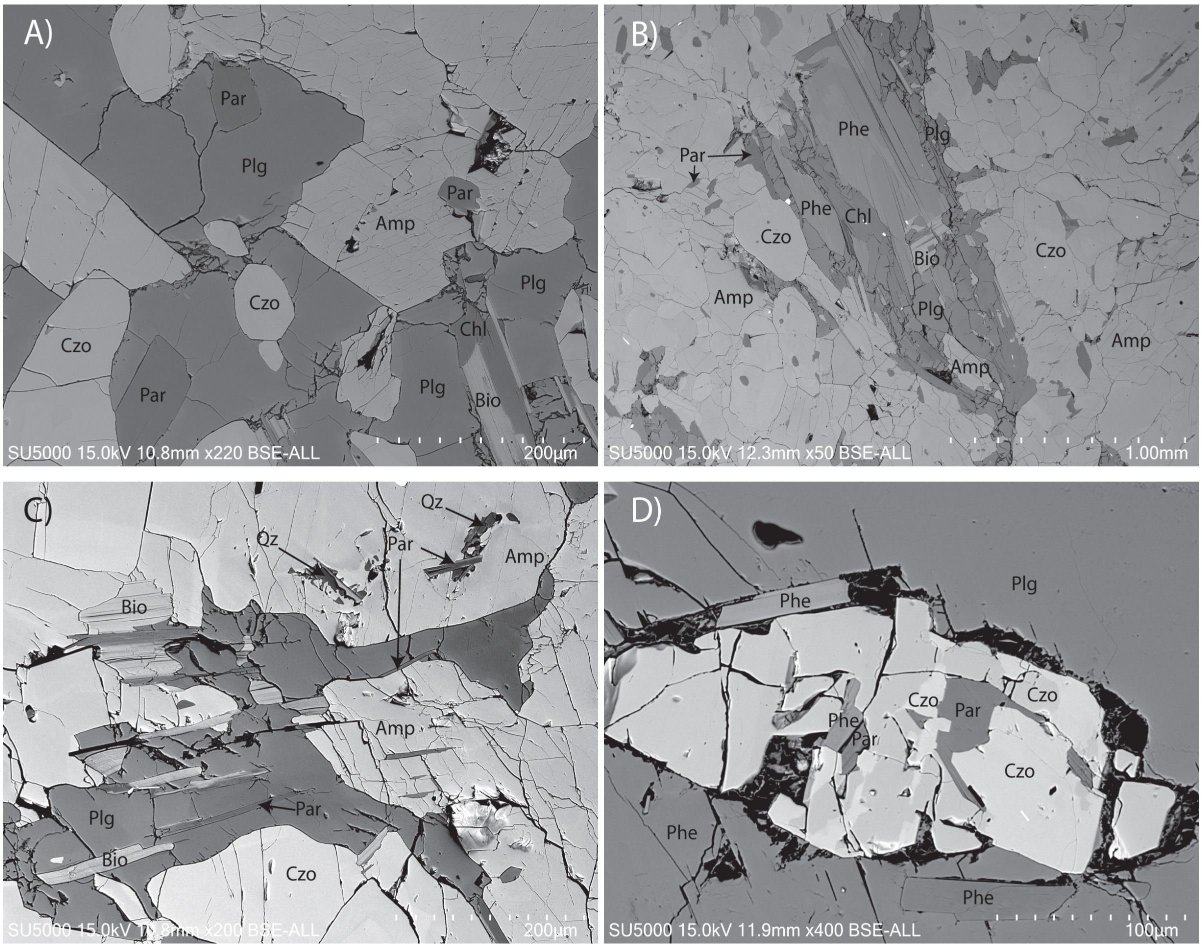

Figure S3 | Back scattered electron (BSE-) images of amphibolite formed from granulite in contact with albite-rich pegmatite. Images show coexisting amphibole (Amp), clinozoisite (Czo), plagioclase (Plg), biotite (Bio), paragonite (Par), phengite (Phe) and rare quartz (Qz).

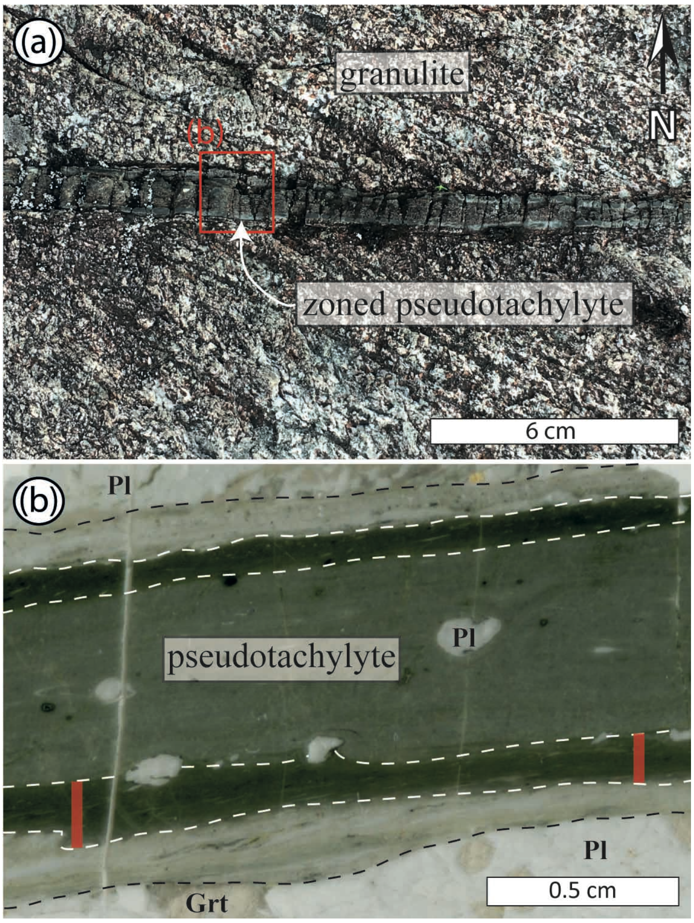

Figure S4 | Pseudotachylyte filled fault in granulite. A) Field image of the large displacement (1.7 m) fault with internally zoned pseudotachylyte. B) Microphotograph of the zoned pseudotachylite. A central light colored zone contains clasts of granulite facies plagioclase (Pl). The homogeneous dark zone (denoted by two vertical red bars) contains plagioclase, biotite, amphibole, clinozoisite, dolomite and garnet and has been used to estimate the conditions under which the pseudotachylite recrystallized (Fig. 2). An outer zone of ultramylonite separates the dark zone from the garnet (Grt) and plagioclase dominated wall rock.

U-Pb data

Table S1. *U-Pb data for pegmatite sample BH-17-007*

	Characteristics*	Weight†,§ [μg]	Pb†,§ [ppm]	U†,§ [ppm]	Th/U‡	Pbc§) [pg]	^{206}Pb/ ^{204}Pb¤	^{207}Pb/ ^{235}U¶	± 2σ [abs]	^{206}Pb/ ^{238}U¶	± 2σ [abs]	rho	^{207}Pb/ ^{206}Pb¶	± 2σ [abs]	^{206}Pb/ ^{238}U¶	± 2σ [abs]	^{207}Pb/ ^{235}U¶	± 2σ [abs]
525/4	Z fr [3]	54	0.16	2.6	0.04	1.5	420	0.5299	0.0066	0.06815	0.00027	0.45	0.05639	0.00063	425.0	1.6	431.7	4.3
525/5	Z fr [3]	93	0.19	3.1	0.00	1.4	860	0.5291	0.0034	0.06788	0.00020	0.55	0.05653	0.00031	423.4	1.2	431.2	2.3
532/25	Z fr [8]	84	0.14	2.2	0.04	5.3	164	0.5414	0.0110	0.06782	0.00029	0.21	0.05790	0.00115	423.0	1.7	439.4	7.2
532/26	Z eu [5]	62	0.19	3.0	0.02	2.0	401	0.5299	0.0055	0.06799	0.00025	0.42	0.05653	0.00054	424.0	1.5	431.8	3.7
532/27	Z fr [4]	83	0.08	1.2	0.00	2.5	188	0.5425	0.0121	0.06806	0.00031	0.32	0.05781	0.00123	424.5	1.9	440.1	7.9
532/28	Z fr cld [6]	109	0.14	2.2	0.02	3.2	336	0.5250	0.0057	0.06771	0.00024	0.37	0.05623	0.00057	422.4	1.4	428.5	3.8

*) Z = zircon, all treated by chemical abrasion (CA) and analyzed by ID-TIMS; eu = euhedral; fr = fragment; cld = partially cloudy after CA: [N] = number of grains in fraction

†,§) weight and concentrations are known to better than 10%.

‡) Th/U model ratio inferred from 208/206 ratio and age of sample.

§) Pbc = total common Pb in sample (initial + blank).

¤) raw data corrected for fractionation and blank.

¶) corrected for fractionation, spike, blank and initial common Pb; error calculated by propagating the main sources of uncertainty.

^) D = degree of discordancy
